# Biomarker in Active Surveillance for Prostate Cancer: A Systematic Review

**DOI:** 10.3390/cancers13174251

**Published:** 2021-08-24

**Authors:** Cécile Manceau, Gaëlle Fromont, Jean-Baptiste Beauval, Eric Barret, Laurent Brureau, Gilles Créhange, Charles Dariane, Gaëlle Fiard, Mathieu Gauthé, Romain Mathieu, Raphaële Renard-Penna, Guilhem Roubaud, Alain Ruffion, Paul Sargos, Morgan Rouprêt, Guillaume Ploussard, on behalf of the CC-AFU, Cancerology Committee of the Association Française d’Urologie

**Affiliations:** 1 Department of Urology, CHU-IUC Toulouse, F-31000 Toulouse, France; 2 Department of Pathology, CHRU Tours, F-37000 Tours, France; gaelle.fromont-hankard@univ-tours.fr; 3 Department of Urology, La Croix du Sud Hospital, F-31130 Quint Fonsegrives, France; jbbeauval@gmail.com (J.-B.B.); g.ploussard@gmail.com (G.P.); 4 Department of Urology, Institut Mutualiste Montsouris, F-75014 Paris, France; Eric.Barret@imm.fr; 5 Department of Urology, CHU de Pointe-à-Pitre, University of Antilles, University of Rennes, Inserm, EHESP, Irset (Institut de Recherche en Santé, Environnement et Travail)–UMR_S 1085, F-97110 Pointe-à-Pitre, France; laurent.brureau@chu-guadeloupe.fr; 6 Department of Radiation Oncology, Curie Institute, F-75005 Paris, France; gilles.crehange@curie.fr; 7 Department of Urology, Hôpital Européen Georges-Pompidou, APHP, Paris–Paris University–U1151 Inserm-INEM, Necker, F-75015 Paris, France; dcharlie8@hotmail.com; 8 Department of Urology, Grenoble Alpes University Hospital, Université Grenoble Alpes, CNRS, Grenoble INP, TIMC-IMAG, F-38000 Grenoble, France; gaellef@gmail.com; 9 AP-HP Health Economics Research Unit, INSERM-UMR1153, F-75004 Paris, France; mathieugauthe@yahoo.fr; 10 Department of Urology, CHU Rennes, F-35033 Rennes, France; romain.mathieu@chu-rennes.fr; 11 Department of Radiology, Sorbonne University, AP-HP, Pitie-Salpetriere Hospital, F-75013 Paris, France; raphaele.renardpenna@gmail.com; 12 Department of Medical Oncology, Institut Bergonié, F-33000 Bordeaux, France; G.Roubaud@bordeaux.unicancer.fr; 13 Service d’Urologie Centre Hospitalier Lyon Sud, Hospices Civils de Lyon, F-69002 Lyon, France; ruffion.alain@orange.fr; 14 Equipe 2–Centre d’Innovation en Cancérologie de Lyon (EA 3738 CICLY)–Faculté de Médecine Lyon Sud–Université Lyon 1, F-69002 Lyon, France; 15 Department of Radiotherapy, Institut Bergonié, 33000 Bordeaux, France; p.sargos@bordeaux.unicancer.fr; 16 Department of Urology, Sorbonne University, GRC 5 Predictive Onco-Uro, AP-HP, Pitie-Salpetriere Hospital, F-75013 Paris, France; mroupret@gmail.com; 17 Institut Universitaire du Cancer Oncopole, F-31000 Toulouse, France

**Keywords:** prostate cancer, biomarker, active surveillance, tissue biomarker, blood biomarker, urine biomarker

## Abstract

**Simple Summary:**

Recently, biomarkers have become a supplemental tool to aid in the diagnosis and evaluation of prostate cancer. Numerous biomarkers are being developed, but no one has a place in current clinical practice for active surveillance. However, active surveillance is a curative treatment option that shifts the possible timing of treatment, but misclassification and progression impose regular monitoring of patients that could be improve by biomarkers. The aim of this review is to investigate the potential of biomarker performance for active surveillance selection and outcome prediction. Our study has identified the critical role that biomarkers could play in piecing together an individualised prognostic for each patient and their use in active surveillance. Although no single biomarker should determine therapy, each biomarker should be considered as a piece of the puzzle in the important decision-making process.

**Abstract:**

Active surveillance (AS) in prostate cancer (PCa) represents a curative alternative for men with localised low-risk PCa. Continuous improvement of AS patient’s selection and surveillance modalities aims at reducing misclassification, simplifying modalities of surveillance and decreasing need for invasive procedures such repeated biopsies. Biomarkers represent interesting tools to evaluate PCa diagnosis and prognosis, of which many are readily available or under evaluation. The aim of this review is to investigate the biomarker performance for AS selection and patient outcome prediction. Blood, urinary and tissue biomarkers were studied and a brief description of use was proposed along with a summary of major findings. Biomarkers represent promising tools which could be part of a more tailored risk AS strategy aiming to offer personalized medicine and to individualize the treatment and monitoring of each patient. The usefulness of biomarkers has mainly been suggested for AS selection, whereas few studies have investigated their role during the monitoring phase. Randomized prospective studies dealing with imaging are needed as well as larger prospective studies with long-term follow-up and strong oncologic endpoints.

## 1. Introduction

Prostate cancer (PCa) is the second most frequent cancer and the fifth leading cause of cancer death in men worldwide [1]. Prostate specific antigen (PSA), FDA approved in 1986 as a prognostic marker in PCa, revolutionized PCa screening with consequent reduction in mortality rates due to diagnosis at earlier stage. However, PSA is characteristic of prostate epithelium but not a cancer specific marker, PSA could be elevated in PCa but in non-malignant condition as benign prostatic hypertrophy or prostatitis. Early detection of PCa by prostate specific antigen (PSA) testing is controversial, with the aim of increasing the detection of localized PCa in order to decrease mortality related to prostate cancer [2]. Two large trials evaluating early detection have reported different results. The PLCO trial conducted in the USA, has shown a higher incidence of PCa but no reduction in PCa mortality [3], but the control arm was contaminated [4]. The more robust European lead ERSPC trial done in Europe, has proven that organized PSA screening provided a PCa specific survival benefit [5]. Over the last few years, we have distinguished between patients with clinically significant PCa and less aggressive PCa that can benefit of active surveillance (AS). AS is a curative treatment option [6,7,8] that shifts the possible timing of treatment while remaining within the window of disease curability and avoids unnecessary aggressive treatment with life-altering side effect [9,10]. The ProtecT study compared surgery, radiotherapy and AS for low and intermediate PCa with no difference between the three approaches confirming the curative possibility by using AS after ten years of median follow up [11,12]. Nevertheless, the criteria for selecting patients eligible for AS and surveillance modalities vary between studies. Overall, criteria are based on digital rectal examination (DRE), PSA or PSA density, ISUP grade and the tumoral burden on the biopsies (number of positive biopsies, percentage of invasion per core). Multiparametric magnetic resonance imaging (mpMRI) has good sensitivity for the detection and location of ISUP grade > 1 [13,14], improves evaluation of PCa with targeted biopsies and could limits the risk of misclassification [15,16].

Misclassification and progression impose regular monitoring of patients in AS. Surveillance frequencies vary between studies but systematically include PSA monitoring, DRE, and repeat biopsies. Repeated biopsies may associated with significant morbidity [17] and non-compliance in men on AS [18]. mpMRI is recommended [6,7] before a confirmatory biopsy but it remains unclear if regular repeat mpMRI should be performed systematically [19,20]. PSA testing reduces death from prostate cancer, but has limited specificity for detecting clinically significant disease. PSA is characteristic of prostate epithelium but not a cancer specific marker, PSA could be elevated in PCa but in non-malignant condition as benign prostatic hypertrophy or prostatitis.

Recently, biomarkers have become a supplemental tool to aid in the diagnosis and evaluation of localized PCa and could allow a more personalized approach to tailor the surveillance of each man. Currently, biomarkers do not yet have a place in current clinical practice for AS.

The aim of this review is to investigate the potential of biomarker performance for active surveillance selection and outcome prediction.

## 2. Methods

A systematic web search was performed according to the Preferred Reporting Items for Systematic Reviews and Meta-analyses (PRISMA) guidelines through the PubMed and Cochrane databases was performed from 2010 to March 2021. A systematic web search was performed according to the Preferred Reporting Items for Systematic Reviews and Meta-analyses (PRISMA) guidelines An ‘a priori’ protocol was submitted to PROSPERO, Registration Number: CRD42021253332.

A systematic web search was performed according to the Preferred Reporting Items for Systematic Reviews and Meta-analyses (PRISMA) guidelines, various algorithms, including the following terms, were used: prostate cancer, active surveillance, localized prostate cancer, biomarker, genomic score, liquid biomarker. Full-text publications using Roman alphabet were considered.

The study population comprised male patients with histologically proven PCa in AS cohort or eligible for AS. We included published full articles, clinical trials, prospective studies and retrospective series, written in English. We excluded retrospective studies with less than 100 patients, abstracts and congress communications. For tissue biomarkers only commercially available biomarkers were studied. Each identified article was analysed and classified.

Main objectives were biomarker performance for active surveillance selection and oncological outcome prediction. Biomarker performance was assessed by their ability to identify grade reclassification (upgrading) or to improve models to identify grade reclassification. They could be used alone but was most often included in models including other criteria.

Risk of bias and study quality was assessed according to EAU recommendations for performing systematic reviews and meta-analysis [21]. The Cochrane risk of bias assessment tool was used for RCTs and the Quality Appraisal tool for case series using a Modified Delphi technique for retrospective studies (Table S1).

**Figure 1:**
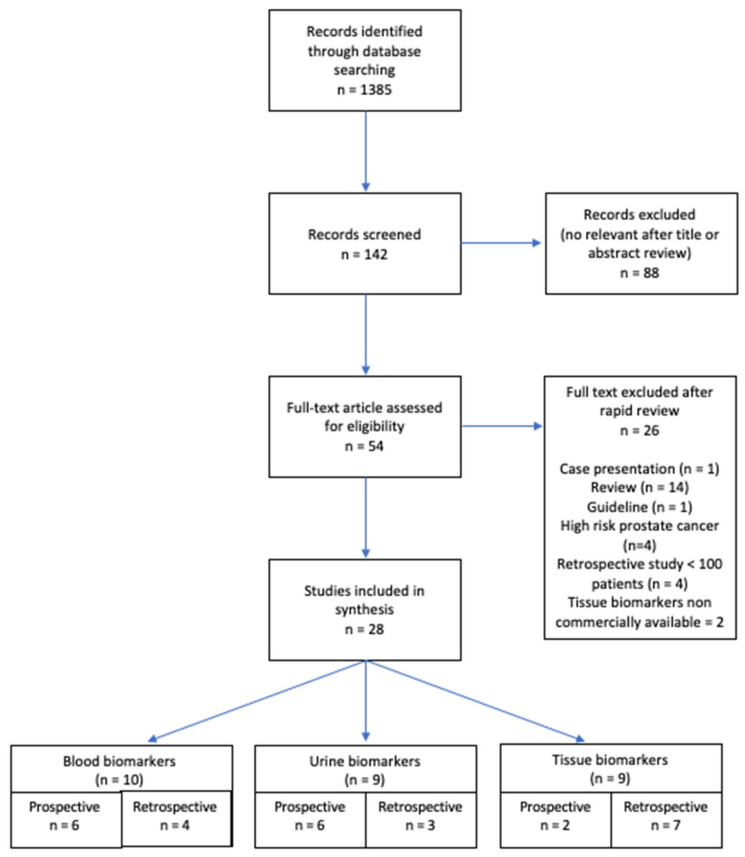
Literature search and selection of studies flowchart.

Selection of article is shown in a flow diagram (Figure 1). Articles were separated for the synthesis into three categories: blood biomarkers, urinary biomarkers, tissue biomarkers.

## 3. Results

A total of 28 articles were selected, of which 10 have a focus on blood biomarkers, 9 on urine biomarkers and 9 on tissue biomarkers.

### 3.1. Blood Biomarkers

Blood biomarkers represent non- or minimally invasive tests which may not be influenced by tumor sampling inherent to prostate needle biopsies.

At the present time, in addition to the PSA, the FDA approved 2 blood biomarkers only for the diagnosis of PCa; the Prostate Health Index (PHI) test and the four-kallikrein (4KScore) score test. Our research has identified 8 biomarkers (Table 1).

### 3.2. ProPSA and Prostate Health Index (PHI)

ProPSA is an enzymatic inactive form of PSA. PSA is secreted as an inactive proenzyme (proPSA) into seminal fluid and activated by the kallikrein-related peptidase 2 and other endopeptidases. In normal situation proPSA do not diffuse into peripherical circulation. In prostate cancer, loss of basal cells, disordering of the basement membrane, and disruption of normal lumen architecture leads to a decrease in luminal processing and a relative increase in bound PSA and proPSA as well as other serous PSA isoforms [32]. Retrospective studies have identified (−2)proPSA as a superior predictor of significant prostate cancer than PSA [33,34,35]. The Prostate Health Index (PHI) is a calculated factor improving the performance of proPSA by combining PSA, freePSA and (−2)proPSA, differentiating the presence of significant PCa from noncancerous prostatic disease [22,36,37]. The test costs about USD 100.

Tosoian et al. [23] examined the relationship between proPSA, PHI and biopsy results in men enrolled in an AS program in a retrospective study including 167 patients with NCCN very low risk PCa. Baseline and longitudinal proPSA and PHI measurements were significantly higher among who presented biopsy reclassification. Heidegger’s et al. [24] international multicenter prospective study, including patients (*n* = 112) with Gleason Score (GS) 6, yet only 44 patients meeting criteria for AS, showed that proPSA and PHI predicted aggressive pathology in univariate analysis but not in multivariate analysis.

More recently, Schwen et al. [38] combined the PHI and mpMRI to predict biopsy reclassification among 253 patients with NCCN low-risk or very low-risk PCa in a retrospective study. In this study PHI and mpMRI would have avoided nearly 20% surveillance biopsies. The inclusion of PHI in the Epstein or PRIAS model increased the accuracy of predicting non-significant PCa, and selecting patient eligible for AS [39,40].

PHI could therefore become a tool for the selection and follow-up of patients in AS, and combined with mpMRI could improve AS and decrease repeated biopsies.

### 3.3. The Four-Kallikrein Panel

The four-kallikrein algorithm was developed based on data from European Randomized Study of Screening for Prostate Cancer (ERSPC) studies and the Prostate Testing for Cancer and Treatment (ProtecT) study [41,42].

Four kallikreins (total PSA, free PSA, intact PSA, and human kallikrein 2) (4K panel) combined with age using a mathematical algorithm gives the 4KScore. The test costs about USD 750.

This score guides urologists’ decisions on whether to perform a biopsy by giving a measure of the probability of significant PCa (GS > 6) for each patient pre-biopsy. This score is known to have a good diagnostic performance in detecting significant PCa [25] and is FDA-approved. Lin et al. [43] explored the utility of the 4K panel to predict high-grade disease in men already diagnosed with GS 6 cancer and on active surveillance in a prospective study (*n* = 718). They used the 4K panel in a different model from the commercial 4K score; the new model included the 4K panel and clinical information available after a diagnosis of cancer, calibrated to an active surveillance population. Replacing the PSA with the 4K panel significantly improved the accuracy for predicting reclassification in the initial surveillance biopsy but there was no benefit for subsequent biopsies. These results should be validated in another larger cohort, but the 4K panel could be a new useful tool for selecting AS candidates.

### 3.4. IsoPSA

IsoPSA is a new blood-based assay for detection of PCa. IsoPSA is a structure-based (rather than concentration-based) assay that interrogates the entire spectrum of structural changes of complex PSA calculated by the equation, K = [([total PSA]bottom − [freePSA]bottom)/([total PSA]top − [free PSA]top)]. Recent studies reported the clinical performance for the detection of high grade (GS > 6) disease [44,45], however no study on AS cohort or low risk population have yet been performed.

### 3.5. Circulating Prostate Cells

Circulating tumoral cells (CTC) is a new simple and less invasive diagnostic concept to identify and investigate the molecular features of solid tumors when cancer cells disseminate into the blood circulation. Detection of CTC in prostate cancer remains in the field of research and is not yet performed in clinical practice.

Most studies looked for an association between CTC count and survival in metastatic PCa [26,46,47] with discordant results using different methods of detection. Murray et al. [27] investigated about the diagnostic performance of malignant prostatic cells detection in blood for early detection of PCa and found sensibility, specificity and negative predictive value of 86.2%, 90.8% and 94.3%, respectively. In another prospective study including 1123 patients referred for prostate biopsy (suspicious DRE or elevated PSA) [48], the authors found that patients with PCa and negative CTC had low grade, small volume tumors and most often would comply with the criteria for active surveillance. Comparison of the presence of CTCs with the clinical pathological findings after RP in men fulfilling the criteria for active surveillance confirmed that positive CTCs represent a high risk of disease upgrade, thus these men may not be ideal candidates for AS [49].

The works of Murray’s team seem interesting for the selection of candidates for AS, but we know that detection of CTCs is highly method dependent and further studies with larger populations are needed.

### 3.6. microRNA (miRNA)

MiRNA circulating in peripheral blood of patient, is a short noncoding RNA that regulate gene expression via modulation of specific messenger RNA (mRNA) targets [50]. In recent years, multiple circulating miRNAs have been shown to be associated with PCa progression or predictive of the response to therapy in high grade or metastatic PCa [28,50]. Liu et al. [51] investigated whether miRNA aberrations are detectable during the early stages of PCa and enables the differentiation from indolent to aggressive PCa. They presented a retrospective description cohort (*n* = 196) and a prospective validation cohort (*n* = 133); all patients had GS 6 and were enrolled in AS program. Three miRNA were significant to predict classification (miR-223, miR-24 and miR-375) and were combined in a 3 mi-RNA score. This study is the first to investigate circulating miRNAs to predict reclassification in patients on AS, but further validations are required.

### 3.7. Caveolin-1

Caveolin-1 (Cav-1), is a membrane protein involved in binding, localizing and regulating of various signalling proteins [52]. The carcinogenic role of caveolin-1 has been identified in many tumors, suggesting that it may act as a novel therapeutic target for tumors. Caveolin-1 is reportedly overexpressed in prostate cancer and could serve as a risk factor and adverse clinicopathological feature of PCa [29]. Basourakos et al. [53] evaluated Cav-1 performance as a biomarker for reclassification in men undergoing AS in a retrospective study (*n* = 542). Baseline Cav-1 level were significantly associated with disease reclassification.

This study suggests that Cav-1 levels may improve risk stratification for AS patients.

### 3.8. Testosterone

Some studies determined a relationship between androgens levels and PCa. Lower serum testosterone concentrations were associated with high-grade PCa, extraprostatic disease and early biochemical recurrence in localized PCa [30,54,55]. Ferro et al. [56] evaluated the association of circulating testosterone concentrations with a reclassification in a cohort of low-risk PCa patients meeting the inclusion criteria for the AS protocol but opting for radical prostatectomy in a restrospective study (*n* = 338). In accordance with previous studies, lower testosterone concentrations were associated with reclassification, disease upgrading and upstaging. Lower testosterone level could help to identify patients at high risk of reclassification and therefore poor candidates for AS.

### 3.9. Stockholm3 Test

The Stockholm3 score predicts the probability of GS > 6 on systematic and targeted biopsy using a combination of 5 plasma biomarkers (total PSA, free PSA, hK2, Macrophage inhibitory cytokine-1 [MIC-1], microseminoprotein-beta [MSMB]), 101 germline genetic markers, and 5 clinical variables (age, first-degree family history of PCa, a previous biopsy, DRE, and prostate volume assessed by transrectal ultrasound at PCa diagnosis) [31,57]. Olsson et al. [58] evaluated this model in AS prospective cohort (*n* = 280). The main outcome was reclassification to GS > 6 and clinically significant PCa. Adding the Stockholm3 test as a selection tool before mpMRI increased sensitivity by 27% to detect GS > 6 and by 53% to detect clinically significant PCa compared with performing systematic biopsies on all men. Of the men with negative Stockholm3 test, 7.9% harboured GS7 (3 + 4) but fewer than 50% of cores were positive and none were clinically significant PCa based on NCCN. These results suggested that Stockholm3 score could decrease the number of MRI investigations needed and biopsied men, whereas missing a small number of significative PCa. However, despite the more frequent use of mpMRI in AS protocol, the Stockholm3 test demonstrates a benefit for AS populations.

## 4. Urinary Biomarker

Urinary biomarkers are other minimally or non-invasive tests, all of which should be performed after DRE. At the present time, the FDA approved PCA3. Our research has identified 4 biomarkers (Table 2).

### 4.1. Prostate Cancer Antigen 3 (PCA3)

PCA3 is a prostate-specific noncoding mRNA detectable in urine. It is significantly over expressed in prostate cancer tissue and tends to be over-expressed in the urine of men with PCa [66,67]. The PCA3 score is a ratio between PCA3-mRNA and PSA-mRNA [68]. PCA3 can be used to risk-stratify men with elevated PSA levels who should undergo a biopsy [69,70]. Higher urinary PCA3 levels were noted in men with higher volume and higher grade PCa [59]. 

The yield of PCA3 in AS protocols is debatable; Tosoian et al. [60] found that PCA3 score was not significantly associated with progression in AS programs in a prospective study (*n* = 293), while Ploussard et al. [61] in another prospective study on low-risk PCa patients (*n* = 106), showed that a PCA3 score > 25 was an important predictive factor for significant PCa and could improve the selection for AS. Later, Tosoian et al. and Newcomb et al. [63,71] in a longitudinal study confirmed that PCA3 scores were significantly higher in men who underwent progression but the change in PCA3 over time was not associated with progression. The inclusion of PCA3 in the Epstein or PRIAS model increased the accuracy of predicting non-significant PCa, and selecting patient eligible for AS [39]. Porpiglia et al. [40] studied PCA3 the performance capabilities of the PHI, PCA3 and mpMRI in predicting the presence of pathologically confirmed significant PCa in patients eligible for AS, but PCA3 did not add value to base model.

According to these studies, PCA3 could be a tool to improve selection for AS but not for follow-ups of AS patients.

### 4.2. TMPRSS2-ERG Fusion

Approximately half of Caucasian patients with PCa over-expressed TMPRSS2:ERG fusion. TMPRSS2:ERG fusion is a rearrangement of the TMPRSS2 gene, an androgen-regulated transcriptional promoter, and the ERG oncogene [72]. TMPRSS2:ERG rearrangement can be detected in urine after DRE and can also be normalized to the amount of PSA mRNA to generate a TMPRSS2:ERG score. Detecting TMPRSS2:ERG fusion in urine was found to be associated with PCa detection, mortality and with tumor volume, and high GS [62].

Lin et al. [73] reported a correlation between urinary levels of combined PCA3 and TMPRSS2:ERG transcripts with aggressive cancer features as defined by tumor volume or Gleason score at the time of diagnosis in a multi-institutional, prospective active surveillance cohort (*n* = 413), but the increase in the markers was not significant. In contrast, the Newcomb et al. study [71] that failed to find an association between TMPRSS:ERG score and disease reclassification in a larger and a newer cohort (*n* = 782).

The relevance of the score is yet to be proven and does not seem to provide more benefit than the PCA3.

### 4.3. DNA Methylation and miRNA

Epigenetic alterations, include DNA methylation and microRNAs (miRNAs), dysregulated in CaP [64,74]. These changes are stable and can be detected in urine. Urinary detection of methylation biomarkers allows for global and non-invasive sampling, which is not the case with biopsies.

Eight genes (APC, CRIP3, GSTP1, HOXD3, HOXD8, KLK10, TBX15 and TGFb2) detected in radical prostatectomy samples were associated with high grade tumors and an adverse clinical prognosis [74], these genes have been looked for in urine sample.

A prospective study has investigated the predictive value of methylation biomarkers in urine samples from patients with PCa enrolled in an AS cohort (*n* = 153) [65]. A 4-gene methylation classifier panel (APC, CRIP3, GSTP1 and HOXD8) was identified and was able to predict patient reclassification.

Another study examined the combination of cell-free urinary miRNA and urinary sediment DNA methylation to develop a multiparametric model for predicting AS patients’ risk reclassification [75]. The authors identified a three-marker panel (CRIP3 methylation, miR-24, and miR-30c) that was a significant predictor for patient reclassification.

These preliminary studies represent a new direction but needs more investigations and validations.

## 5. Tissue Biomarker

All patients included in AS protocol have diagnosis biopsies and repeated monitoring biopsies. Tissular biomarkers have been developped to predict the prognosis of the disease by studying PCa cells. Currently 4 biomarkers are commercially available in some countries: Oncotype Dx GPS©, Decipher©, Prolaris© and Promark score©. The cost ranges from USD 3000 to USD 5000, but can be covered by commercial insurance.

Important studies are resumed in Table 3.

### 5.1. Oncotype Dx Genomic Prostate Score©

The OncotypeDx Genomic Prostate Score is a RNA based expression assay of 12 PCa related genes normalized to 5 housekeeping genes. Quantitative reverse transcriptase-polymerase chain reaction assay is performed, and the genomic expression levels are used in an algorithm, the “genomic prostate score (GPS)“, in which different gene clusters are given different weight (those involved in the androgen pathway are given higher weight than others). The GPS ranges from 0 to 100 with higher scores indicating a greater genomic risk of aggressive disease. The assay is available in the US at a single platform. GPS can be performed on prostate specimens or on needle core biopsy tissue with more than 1 mm prostate tumor. It has been clinically validated to predict the risk of high grade and/or non-organ confined disease, and time to biochemical recurrence and metastasis [76,85,86].

Several retrospective studies [77,78,79] and one prospective study [76] characterized GPS in patients eligible for AS. A higher initial GPS was associated with an increased risk of upgrading, and an increased risk of adverse pathology [76,77,78,79]. For monitoring, the GPS is relatively stable with time even in men with a biopsy upgrade [79]. Some studies associated GPS and mpMRI, one associating PI-RADS and GPS on systematic biopsies [77], another associating PIRADS and GPS on targeted and systematic biopsies [80]. In these studies, the GPS score was predictive of adverse disease pathology on the prostate specimen, independently of the MRI scores.

However, most recently, a multicentre prospective-retrospective study evaluated the performance of GPS in patients on AS (*n* = 432) [81]. This study found that GPS scores were not associated with adverse pathology at RP nor upgrade on subsequent biopsy. Moreover, Nyame et al. [82], studied GPS in patients with very low- and low-risk NCCN PCa, and did not demonstrate any GPS differences with disease volume at prostate biopsy.

These inconsistencies make it impossible to conclude on the use of GPS for the selection of patients in AS. Prospective validations of this marker are awaited.

### 5.2. Genomic Classifier: Decipher©

Decipher uses a whole-transcriptome microarray assay. A total of 22 RNA biomarkers (coding and non-coding) selected by machine learning have been associated with PCa aggressiveness and metastasis prediction after RP [87]. An algorithm generates a score ranging from 0 to 1 with higher values indicating poor prognosis. The assay is available in the US at a single platform.

This test was performed on RP specimens, but recently results have been reported for the analysis of biopsy needle, however without defined pre-analytical conditions.

Recent review, showed the utility of Decipher© for intermediate-risk PCa and postprostatectomy decision-making and was prognostic for adverse pathology, biochemical failure, metastasis, and cancer-specific and overall survival [88].

Kim et al. [83] and Herleman et al. [84] evaluated the ability of Decipher© to predict adverse pathology in patients with NCCN favourable-intermediate risk PCa who undergone a RP at first treatment. Decipher was an independent predictor of adverse pathology and adding Decipher improved the CAPRA score. In this setting, Decipher©-high-risk patients were not good candidates for AS. Decipher© test could be a tool for patient selection but need more prospective studies.

### 5.3. Prolaris©

Prolaris evaluates genes related to cell cycle progression. The assay uses RT-PCR to look at expression levels of 31 genes involved in the cell cycle progression pathway and 15 housekeeper genes. The expression level of these genes is included in an algorithm that calculates a score, the CCP, which is a continuous variable between −3.0 and 7.0 with higher value indicating poor prognosis.

This test has been reported mainly on prostatectomy specimens, when performed on biopsy material, the test requires a tumour length of more than 2 mm. The assay is available in the US at a single platform.

It has been validated as an independent prognostic factor after RP in a cohort including low risk PCa [89] but since then no studies have been conducted on an AS cohort.

### 5.4. ProMark Score©

ProMark score use a quantitative multiplex proteomics in situ imaging system to identify and measure 8 protein-based biomarkers that are able to predict prostate cancer aggressiveness and lethal outcome. The ProMark score^®^ ranges from 0 to 100 with higher score relating to more aggressive and lethal PCa [90]. It was initially developed on RP specimen and then on biopsy material. No pre-analytical criteria are reported for the minimum amount of tumour tissue required.

This score offered additional prognostic value for individual patients relative to NCCN risk categories alone. No supplementary study was carried out.

## 6. Discussion

With the heterogenous nature of prostate cancer, it is essential to identify optimal methods to guide physicians in selecting the best personalised treatment for their patients. Whilst AS aims to avoid unnecessary invasive treatment in men with localised PCa and simultaneously, examines and enables selection of patients needing further treatment in an ideal curative time window, the mode of AS could be improved. The recent development of numerous biomarkers could become a novel tool to improve PCa risk assessment and contribute to patient wellbeing. One study showed that among patients with PCa cancer at favourable risk, those classified as low risk using a biomarker were more likely to be managed on AS than those who had not been tested [91].

Table 4 lists the biomarkers performance presented in the article.

Serum and urine biomarkers are non- or minimally invasive tests, less invasive than repeated biopsy. Currently, PHI, 4KScore and PCA3 are FDA approved and seem to be of prognostic interest for the selection of AS-eligible patients. PHI also appears to be relevant for AS monitoring. Of note, the urinary biomarkers could reduce morbidity and facilitate compliance in men on AS. Moreover, these biomarkers are not influenced by tumour sampling which suggests a greater stability in the assays with a global disease assessment. Most of the other liquid biomarkers (circulating prostate cells, microRNA, Caveolin 1) seem like attractive tools for the selection of patients eligible for AS. The disease monitoring was less evaluated on AS although it represents the main challenge for these population and therefore studying disease progression is of significance.

Genomic scores represent new area in PCa, predicting prognosis by studying PCa cells. The limitations of these tests are tumour sampling and the minimum amount of tissue material required inherent to prostate needle biopsies but represent the only way to study tumour cell directly. Of the four tissular biomarkers commercially available, OncotypeDX GPS© and Decipher© have been studied in AS population and may become new helpful tools for AS selection in the case where tissue is available. Many genomic score are in development, but tumour heterogeneity and sampling fluctuations require large robust studies.

Biomarkers are of recent development in PCa and there are many limitations. They require increasingly complex technologies, and a lot of them are not yet available in clinical routines, which explains the low number of studies.

The majority of biomarkers recently published are still in the investigation or validation phases and AS population have not yet been studied. Furthermore, heterogeneity regarding study design and population characteristics could explain the discrepancy in results. No data is yet available with long term endpoints such as disease mortality in patients with more than 10 years life expectancy and a disease leading specifically to death on long-term follow-up. Finally, since most studies are retrospective, prospective studies investigating the performance of biomarkers in PCa have yet to be conducted. Additionally, biomarkers are currently expensive, the cost effectiveness of biomarkers has not been studied and is poorly described in literature but remains a key question in the management of patients.

## 7. Conclusions

Numerous biomarkers are being developed, which could become novel tools to improve PCa risk assessment and contribute to patient wellbeing. At present, it is difficult to conclude and establish recommendations. Nonetheless, biomarkers hold an exciting prospect as a new prognostic strategy that would make it possible to offer personalized medicine, individualizing the treatment and monitoring of each patient. Further larger prospective studies with long term outcomes are required to define how these novel biomarkers could be used to select men that would most benefit from an AS program and how these markers could be incorporated into the follow-up schedule of AS patients. Studies combining biomarkers with mpMRI data, known to be a strong diagnostic and prognostic tool, seem indispensable to be adapted to current practices. Combining the use of different biomarkers would provide greater understanding of each disease type, opening new avenues of research and prognostic options. Finally, despite a lack of research carried out with a specific focus on biomarkers, our study has identified the critical role that biomarkers could play in piecing together an individualised prognostic for each patient and their use in AS. Although no single biomarker should determine therapy, each biomarker should be considered as a piece of the puzzle in the important decision-making process.

## Figures and Tables

**Table 1:** Blood biomarkers.

Biomarker	Article	Objective	Population	Results	Conclusion
**ProPSA and Prostate Health Index (PHI)** *(Serum)*	Tosoian et al. 2012 [22]	To examine the relationship between proPSA and biopsy results in men enrolled in AS program. RETROSPECTIVE STUDY	Patients with NCCN very low risk PCa in AS program (*n* = 167)	37.7% had GR on follow-up. Baseline and longitudinal proPSA, and PHI measurements were significantly associated with biopsy reclassification in Cox models while total PSA was not.	Baseline and longitudinal proPSA and PHI measurements were significantly higher among men in AS surveillance who had GR *SELECTION OF CANDIDATE FOR AS AND MONITORING*
**ProPSA and Prostate Health Index (PHI)** *(Serum)*	Heidegger et al. 2017 [23]	To evaluate the impact of PSA isoforms on risk stratification in patients with low-risk PCA as well as in AS candidates who underwent RP PROSPECTIVE STUDY	Patient with GS 6 PCa scheduled for RP (*n* = 112) 44 patients met the criteria for active surveillance (AS) according to the EAU and NCCN criteria	66.7% of patients had a GR. proPSA outperformed PSA and freePSA in predicting aggressive PCa (GS upgrading and adverse pathology) as well as positive margins PHI has an even higher predictive power when compared with proPSA alone concerning GR (*p* = 0.004), extraprostatic extension (*p* < 0.001) and surgical margins (*p* = 0.051). Not emphasize any of the factors to influence significantly the outcome of the findings in a multivariate context.	ProPSA and PHI predict aggressive pathology in univariate analysis but not in multivariate in GS 6 PCa. *SELECTION OF CANDIDATE FOR AS*
**PHI** *(Serum)*	Schwen et al. 2020 [24]	To identify the value of combining the PHI and mpMRI, for the purpose of GR at the next surveillance biopsy in PCa AS. RETROSPECTIVE STUDY	Patients with NCCN low-risk or very low-risk PCa in AS program (*n* = 253)	15% had GR during surveillance biopsy 1 unit increase in PHI was associated with an OR of 1.02 for GR. Above the 25th percentile cut-off, PHI, PHI density and PSA density were each significantly associated with GR. The combined use of a PHI < 25.6 and PI-RADSv2 ≤ 3 suggests 20% of surveillance biopsies could have been avoided at the cost of missing only 2.6% (one of 38) of GR.	PHI and mpMRI could be used to accurately predict GR in men on AS in our cohort of low-risk PCa. When used in combination, PHI and mpMRI have the potential to substantially reduce the number of surveillance biopsies. *SELECTION OF CANDIDATE FOR AS AND MONITORING*
**4 kallicrein panel (4Kpanel)** *(Plasma)*	Lin et al. 2017 [25]	To evaluate the utility of a 4Kpanel in predicting the presence of high-grade PCa in men on AS. PROSPECTIVE STUDY	Patients in PASS protocol: Histologically confirmed PCa, ECOG performance status of 0 or 1, clinical T1 - T2 disease, no previous treatment for PCa, enrolled on AS two groups: (1) the initial biopsy after cancer diagnosis (2) all subsequent surveillance biopsies. (*n* = 718) 478 in the initial biopsy group for whom kallikreins were assayed 319 in the training set	ROC curve analysis comparing the full model with the 4Kpanel and the full clinical model with serum PSA indicated that the 4Kpanel significantly improved the accuracy for predicting reclassification (AUC 0.78 vs. 0.74) in the initial surveillance biopsy, with a significant incremental value in AUC. The 4Kpanel did not improve prediction of reclassification in subsequent biopsies relative to PSA (AUC 0.75 vs. 0.76).	Addition of 4Kpanel to a model that includes clinical information can significantly improve prediction of the outcome in the first surveillance biopsy The 4Kpanel was not of value over PSA for the prediction of reclassification in subsequent biopsies after the first surveillance biopsy *SELECTION OF CANDIDATE FOR AS*
**Circulating prostate cells (CPCs)** *(Serum)*	Murray et al. 2014 [26]	To determine if primary CPCs are found in all men with PCa PROSPECTIVE STUDY	Men with PSA between 4.0 and 10.0 ng/mL and/or a DRE suspicious of PCa and were referred for prostate biopsy. (*n* = 1123)	29.2% had positive biopsies, among men with positive biopsies, 12.8% were negative for the detection of CPCs. Men negative for CPCs had lower serum PSA levels, lower Gleason scores, lower number of cores positive for PCa, and cores less infiltrated with cancer. 91% of CPC negative men complied with the criteria for AS of their PCa. whereas only 12% (*p* < 0.0001) of CPC positive men complied with the criteria for AS.	The majority of cancers with CPC negative are low grade small volume tumors which would comply with the criteria for AS. *SELECTION OF CANDIDATE FOR AS*
**Circulating prostate cells (CPCs)** *(Serum)*	Murray et al. 2017 [27]	To compare the presence or absence of primary CPCs with the clinical pathological findings after RP in men fulfilling the criteria for AS. PROSPECTIVE STUDY	Men with a PCa fulfilled the Epstein criteria for AS underwent for RP (*n* = 102)	24.51% were upgraded based on the results of the surgical specimen Men CPCs positive had a frequency of upgrade of 44.44% versus a 8.77% for men CPCs negative, with a difference (*p* < 0.0001) Therefore CPCs positive men showed a relative risk of 5.07 with an absolute risk difference of 35.67% of being upgraded	In men fulfilling the criteria for AS but are positive for primary CPCs detection, there is a high risk of disease upgrade, thus these men may not be ideal candidates for AS. *SELECTION OF CANDIDATE FOR AS*
**microRNA** *(Serum)*	Liu et al. 2018 [28]	To investigate promising circulating miRNA biomarkers to predict the reclassification of AS cases. PROSPECTIVE STUDY	2 independent AS cohorts of 196 (retrospective) for the training and 133 (prospective) for the validation sample Patient diagnosed with GS 6 PCa and enrolled in AS cohort.	*Training*: logistic regression was used to construct a weighted combination of miR-223, miR-24 and miR-375, which was significant to predict reclassification This 3-miR score was a better predictor than any individual miRNA or clinical variable. *Validation*: The 3-miR score was still a significant predictor of reclassification (OR 3.70 95% CI 1.29–10.6) and it outperformed PSA (OR 1.25, 95% CI 1.08–1.44).	The 3-miRNA score can be used in addition to PSA to identify cases that are unlikely to be reclassified. *SELECTION OF CANDIDATE FOR AS*
**Caveolin-1** *(serum)*	Bousarakos et al. 2017 [29]	To evaluate the role of caveolin-1 as a predictor of disease reclassification in men with early PCa undergoing AS. RETROSPECTIVE STUDY	Early PCa in a single-institution AS study (*n* = 542)	30.1% were reclassified. In univariate analysis, the risk of disease reclassification was significantly associated with having a higher baseline Cav-1 level (OR 1.82, 95% CI 1.24–2.65, *p* = 0.002) In the multivariate regression, baseline Cav-1 levels (*p* = 0.001) were significantly associated with disease reclassification.	Baseline plasma caveolin-1 level was an independent predictor of disease classification. *SELECTION OF CANDIDATE FOR AS*
**Testosterone** *(serum)*	Ferro et al. 2017 [30]	To evaluate the association of circulating testosterone concentrations with a staging/grading reclassification in a cohort of low-risk PCa patients meeting the inclusion criteria for the AS protocol but opting for RP. RETROSPECTIVE STUDY	Patients with low risk PCa fulfilled the inclusion criteria for the PRIAS protocol (*n* = 338)	Lower total testosterone levels were associated with upstaging, upgrading, unfavorable disease and predominant Gleason score 4 in prostate specimen. Total testosterone included was a significant independent predictor, both as a continuous and dichotomous variable, of upstaging, upgrading and unfavorable disease. A significant gain in predictive accuracy was only detected for the outcome of upstaging and predominant GS 4. No advantages over the base model were observed for the outcome of upgrading, unfavourable disease and for the prediction of positive surgical margins.	Men with hypogonadism eligible for AS are at higher risk of disease upgrading and upstaging compared to men with normal serum testosterone levels. *SELECTION OF CANDIDATE FOR AS*
**Stockholm3 test** *(Plasma)*	Olsson et al. 2020 [31]	To evaluate an AS protocol using the Stockholm3 test and mpMRI to reduce biopsy intensity. PROSPECTIVE STUDY	GS 3+3, currently on AS, had to be alive without any severe comorbidity, contraindications for MRI, or a history of initiating PCa treatment underwent MRI and prostate biopsy (*n* = 280)	23.3% were reclassified. Adding the Stockholm3 test as a selection tool before MRI increased sensitivity by 27% to detect GS ⩾ 3+4 cancer (RS = 1.27, 95% CI = 1.02 to 1.65) and by 53% to detect significant PCa (RS = 1.53, 95% CI = 1.13 to 2.36) compared with performing systematic biopsy on all men, while decreasing the number of MRI investigations by 22.5% and the number of biopsied men by 56.8% Of the men with negative Stockholm3 test, 7.9% harbored GS ⩾ 3+4 PCa (but less than 50% cores), and no participants with a negative Stockholm3 test had significant PCa according to NCCN.	Stockholm3 test decrease the number of MRI investigations needed and biopsied men. *SELECTION OF CANDIDATE FOR AS AND MONITORING*

4K: 4 kallicrein; AS: active surveillance; CPCs: Circulating prostate cells; DRE: digital rectal examination DRE; GS: gleason score; GR: grade reclassification (upgrading); mpMRI: multiparametric magnetic resonance imaging; NCCN: national comprehensive cancer network; PCa: prostate cancer; PHI: Prostate Health Index; PSA: prostate serum antigen; RP: Radical prostatectomy.

**Table 2:** Urine biomarkers.

Biomarker	Article	Objective	Population	Results	Conclusion
**Prostate cancer antigen 3 (PCA3)** *(Urine after DRE)*	Tosoian et al. 2010 [59]	To assess the relationship between PCA3 and prostate biopsy results in men in AS. PROSPECTIVE STUDY	Patients NCCN very low risk PCa. (*n* = 293)	12.9% had GR. ROC analysis suggested that PCA3 alone could not be used to identify men with progression. Cox proportional hazards model after adjustment for age and date of diagnosis PCA3 was not significantly associated with progression (*p* = 0.15).	Trend toward higher PCA3 scores in patients with GR on biopsy. Overlap in PCA3 levels in comparing those with and those without progression. Unable to identify a threshold value for PCA3
**Prostate cancer antigen 3 (PCA3)** *(Urine after DRE)*	Ploussard et al. 2011 [60]	To assess the impact of urinary PCA3 score as an AS criterion instead of and in addition to the current criteria. PROSPECTIVE STUDY	Patients with NCCN low-risk PCa who underwent a PCA3 urine test before RP. (*n* = 106)	27.4% overall unfavorable disease The mean PCA3 score was higher in patients with significant disease compared with patients with insignificant disease (organ confined, no Gleason pattern 4 or 5, tumour volume < 0.5 cm^3^), according to the Epstein criteria (60.1 vs. 29.3, *p* < 0.001) In a multivariate analysis taking into account each AS criterion (biopsy criteria, PCA3 score, MRI findings, PSA density), a high PCA3 score (>25) was an important predictive factor for significant PCa (OR: 12.7; *p* = 0.003) and for tumour volume ⩾ 0.5 cm^3^ (OR 5.4; *p* = 0.010).	PCA3 score may be a useful maker to improve the selection for AS in addition to the current AS criteria. Trend towards higher PCA3 scores in patients with unfavourable, significant, and large-volume PCa. unable to identify a threshold value for PCA3 that could accurately classify high-risk men with non–organ-confined disease PCA3 score cannot be integrated in AS selection as a single prognostic variable. *SELECTION OF CANDIDATE FOR AS*
**Prostate cancer antigen 3 (PCA3)** *(Urine after DRE)*	Tosoian et al. 2017 [61]	To assess the utility of PCA3 as both a one-time and longitudinal measure in men on AS. RETROSPECTIVE STUDY	Patients with NCCN Very low risk PCa, and NCCN low risk PCa (*n* = 260)	10.8% demonstrated GR. Patients who underwent GR had significantly higher PCA3 scores at both the first (48.0 vs. 24.5, *p* = 0.007) and subsequent (63.5 vs. 36.0, *p* = 0.002) measures. Analysis confirmed in multivariate model. They not demonstrate a significant association between longitudinal increase in PCA3 and subsequent identification of high-grade cancer.	The first and subsequent urinary PCA3 scores were significantly higher in men who underwent GR during follow-up. The change in PCA3 over time was not associated with reclassification. *SELECTION OF CANDIDATE FOR AS*
**Prostate Health Index (PHI) *(serum)* and Prostate** **cancer antigen 3 (PCA3)** *(Urine after DRE)*	Cantiello et al. 2016 [38]	To assess the PHI and PCA3 when added to the PRIAS or Epstein criteria in predicting the presence of pathologically insignificant PCa in patients who underwent RP but eligible for AS. RETROSPECTIVE STUDY	Patients eligible for AS based on PRIAS criteria or Epstein criteria (*n* = 188)	On multivariate the inclusion of both PCA3 and PHI significantly increased the accuracy of the Epstein criteria and PRIAS model in predicting significant PCa after adjusting for age and biopsy GS	Epstein and PRIAS protocols can be improved by the addition of PCA3 or PHI resulting in a greater net benefit in predicting insignificant PCa in men eligible for AS. *SELECTION OF CANDIDATE FOR AS*
**Prostate Health Index (PHI)***(serum)***and Prostate cancer antigen 3 (PCA3**) *(Urine after DRE)*	Porpiglia et al. 2016 [39]	To assess the performance capabilities of mpMRI, PHI and PCA3 in predicting the presence of pathologically confirmed significant PCa in a cohort of patients who underwent RP but who were eligible for AS. RETROSPECTIVE STUDY	Patients with biopsy-proven, clinically localized PCa, eligible for AS based on PRIAS criteria who underwent RP *(n = 120)*	mpMRI showed good specificity and negative predictive value (0.61 and 0.73, respectively) for excluding significant PCa. mpMRI significantly increased the accuracy of the base model in predicting significant PCa by 7%. The PHI significantly increased the accuracy of the base model in predicting significant PCa by 4%. The model that included PCA3 did not add value.	mpMRI and, to a lesser extent, the PHI had an important role in discriminating the presence of significant PCa. *SELECTION OF CANDIDATE FOR AS*
**Prostate cancer antigen 3 (PCA3) and TMPRSS2-ERG mRNA** *(urine after DRE)*	Lin et al. 2013 [62]	To determine whether urinary PCA3 and TMPRSS2-ERG mRNA levels are associated with higher volume or grade PCa in a multi-institutional AS cohort. PROSPECTIVE STUDY	Patients in PASS clinical protocol: Histologically confirmed PCa, ECOG performance status of 0 or 1, clinical T1 - 2 disease, no previous treatment for PCa *(n = 413)*	In univariate analyses both markers appear to stratify for baseline risk of disease aggressiveness as defined by biopsy GS or volume of tumor (% of positive cores). There is a trend towards these biomarkers improving the power of PSA to predict high grade or volume disease, but not significant. Results from multivariable logistic regression models were not significant after adjusting for covariates	PCA3 and TMPRSS2-ERG mRNA appear to stratify risk at time of enrollment, for men on AS, of having aggressive cancer as defined by tumor volume or GS. Multivariable logistic regression were not significant. SELECTION? MONITORING?
**PCA3 and TMPRSS2-ERG mRNA** *(Urine after DRE)*	Newcomp et al. 2019 [63]	To evaluate the association between urinary PCA3 and TMPRSS2-ERG mRNA and biopsy reclassification using urine collected at multiple times during AS. PROSPECTIVE STUDY	Patients in PASS clinical protocol: Histologically confirmed PCa, ECOG performance status of 0 or 1, clinical T1 - 2 disease, no previous treatment for PCa (*n* = 782)	Of the 552 men with urine biomarkers assessed prior to the first surveillance biopsy, 24% were reclassified at that biopsy. In a logistic regression model adjusted for PSA, cores ratio, and prostate size, PCA3 score was associated with reclassification in the first surveillance biopsy (OR = 1.3; 95% CI: 1.0–1.7), and TMPRSS2-ERG mRNA score was not. In a logistic regression model adjusted for clinical variables, neither PCA3 nor T2:ERG were associated with reclassification	Significant association of PCA3 with reclassification at the first surveillance biopsy, but only a modest improvement in AUC between the model with clinical variables only and a model plus PCA3. No association between either baseline PCA3 or TMPRSS2-ERG mRNA and time to reclassification, and no association between changes in the biomarker scores over time and time to reclassification No association between biomarker kinetics and reclassification *SELECTION OF CANDIDATE FOR AS*
**DNA methylation** *(Urine after DRE)*	Zhao et al. 2017 [64]	To investigate the predictive value of methylation biomarkers in urine samples from patients with PCa enrolled in a characterized Canadian AS cohort PROSPECTIVE STUDY	Patient diagnosed with GS 6 PCa and treatment naïve enrolled in AS cohort. (*n* = 153)	22.2% reclassified with higher risk disease. Multivariate logistic regression analysis demonstrated that the classifier panel (the weighted sum of APC, CRIP3, GSTP1 and HOXD8 methylation) was an independent predictor of patient reclassification.	The classifer panel is predictive for patient reclassification in AS cohort. Validation is needed *SELECTION OF CANDIDATE FOR AS AND MONITORING*
**Free miRNA and sediment DNA methylation** *(Urine after DRE)*	Zhao et al. 2019 [65]	To examine the combination of cell-free urinary miRNA and urinary sediment DNA methylation to develop a model for predicting AS patients’ risk reclassification PROSPECTIVE STUDY	Treatment naïve patients diagnosed with GS 6 tumors, cT1-T2, recruited into AS program (*n* = 103)	CRIP3 methylation, miR-24, and miR-30c = the 3-marker panel and was a significant predictor for reclassification In multivariable logistic regression the 3-marker panel was found to be an independently significant predictor.	Integrated urinary 3-marker panel composed of miR-24, miR-30c, and methylation of CRIP3 was able to significantly predict AS patient reclassification. The 3-marker panel correctly identified over 80% of AS patients who will experience reclassification. Validation is needed *SELECTION OF CANDIDATE FOR AS*

AS: active surveillance; CPCs: Circulating prostate cells; DNA: deoxyribonucleic acid; DRE: digital rectal examination; ECOG: Eastern Cooperative Oncology Group; GS: gleason score; GR: grade reclassification (upgrading); mpMRI: multiparametric magnetic resonance imaging; NCCN: national comprehensive cancer network; PCa: prostate cancer; PCA3: Prostate cancer antigen 3; PHI: Prostate Health Index; PRIAS: Prostate Cancer Research International Active Surveillance; PSA: prostate serum antigen; RNA: ribonucleic acid; RP: Radical prostatectomy.

**Table 3:** Tissular biomarkers.

Biomarker	Article	Objective	Population	Results	Conclusion
**Oncotype Dx GPS** *(random biopsy)*	Klein et al. 2014 [76]	To identify and validate a biopsy-based gene expression signature that predicts clinical recurrence, PCa death, and adverse pathology in patients. PROSPECTIVE STUDY	Retrospective study: PCa patients treated by RP with clinical recurrence (*n* = 127) were selected together with a random nonrecurrent patients (*n* = 374) ratio 1:3 Prospective study: PCa patients candidates for AS but elected prostatectomy	Prospective study: 31% had high-grade or non–organ confined disease at prostatectomy GPS was a significant predictor of pathologic stage and grade at prostatectomy, adjusting for biopsy GS (*p* = 0.002). In separate multivariable analyses adjusting for significant clinical covariates, the GPS was a consistent predictor of high-grade and/or non–organ-confined pathology, as were traditional clinical predictors	GPS improves risk stratification at time of diagnosis in patients candidates for AS *SELECTION OF CANDIDATE FOR AS*
**Oncotype Dx GPS** *(random biopsy)*	Kornberg et al. 2019 [77]	To determine whether GPS and PI-RADS score are associated with an increased risk of GR in men on AS. RETROSPECTIVE STUDY	Patients treated with AS for low/intermediate risk PCa who underwent 1 or more surveillance biopsies, and GPS testing and/or mpMRI prior to the upgrade or the last biopsy. MRI and GPS tests were ordered at the discretion of the treating clinicians. *n* = 169 PI-RADS score only *n* = 140 GPS only *n* = 131 GPS and PI-RADS score	The GPS was associated with an increased risk of upgrading. PI-RADS scores of 5 vs. 1–2 and 4 vs. 1–2 were associated with an increased risk of a GR. In patients who undergo mpMRI and the GPS, the GPS is independently associated with GR but the PI-RADS score is not.	A higher GPS score or a PI-RADS score of 4 or 5 was associated with an increased risk of biopsy upgrading. In men with a GPS and a PI-RADS score only the GPS was independently associated with a GR. *SELECTION OF CANDIDATE FOR AS AND MONITORING*
**Oncotype Dx GPS** *(random biopsy)*	Kornberg et al. 2019 [78]	To evaluate the GPS test in men with low or intermediate risk PCa on AS and to determine whether a higher GPS score is associated with an increased risk of adverse pathology and/or biochemical recurrence among men who underwent delayed RP after an initial period of AS. RETROSPECTIVE STUDY	Patients on AS surveillance who had GS 6 or low volume (33% or fewer positive cores) GS 7 (3 + 4) PCA, GPS testing at diagnostic or confirmatory biopsy, clinical stage T1/T2, PSA less than 20 and a clinical CAPRA score less than 6. *n* = 215	On multivariate analysis the GPS was independently associated with an increased risk of adverse pathology at RP. The GPS was independently associated with biochemical recurrence following delayed RP.	In men with low and intermediate risk PCa who enroll in AS and go on to delayed RP a higher GPS at baseline is independently associated with an increased risk of adverse pathology and biochemical recurrence following definitive treatment. *SELECTION OF CANDIDATE FOR AS*
**Oncotype Dx GPS** *(random biopsy)*	Cedars et al. 2019 [79]	To characterize the stability and usefulness of serial GPS in men undergoing serial biopsies during AS. RETROSPECTIVE STUDY	Patients initially diagnosed with GS 6 PCa *n* = 111	A higher GPS at first biopsy was associated with a risk of GR at second biopsy (*p* = 0.03). The GPS at second biopsy was not associated with a GR when added to the base model (*p* = 0.13). In models including only the GPS at first biopsy and only the GPS at second biopsy there was no incremental benefit to including serial scores in a single model. In the base model plus the GPS at first biopsy, the GPS and the GPS difference were associated with a risk of treatment In the base model plus the GPS at second biopsy only the GPS was associated with higher risk of undergoing active treatment.	The GPS undergoes small changes with time. The initial test is the most informative one and serial testing seems to have limited benefit. Absolute GPS results at the first and second biopsies were associated with GR and transition from AS to active treatment. *SELECTION OF CANDIDATE FOR AS AND MONITORING*
**Oncotype Dx GPS** *(systematic and random biopsy)*	Salmasi et al. 2018 [80]	To investigate the ability of the GPS to predict adverse pathology findings in the setting of magnetic resonance imaging guided prostate biopsy RETROSPECTIVE STUDY	NCCN very low, low or intermediate risk prostate cancer patients who underwent simultaneous MRI fusion targeted and systematic prostate biopsy with subsequent RP within 6 months. *n* = 134	GPS was an independent predictor of adverse pathology but MRI score not.	The GPS is an independent predictor of adverse pathology findings in patients who were diagnosed with very low, low or intermediate risk prostate cancer in the setting of MRI fusion prostate and systematic biopsies. *SELECTION OF CANDIDATE FOR AS*
**Oncotype Dx GPS** *(random biopsy)*	Lin et al. 2020 [81]	To examine the association of GPS results with outcomes relevant to AS. PROSPECTIVE-RETROSPECTIVE STUDY	Patients in PASS protocol with low-risk PCa. *n* = 432	In multivariable analysis of the 432 men on AS there was no significant association of GPS with GR. In multivariable analysis of the 101 men who had RP, GPS did not reach statistical significance.	The GPS was not associated with unfavourable disease, and there was no association with GR in surveillance biopsy. Adding GPS did not significantly improve stratification of risk.
**Oncotype Dx GPS** *(random biopsy)*	Nyame et al. 2018 [82]	To determine whether disease volume at prostate biopsy would correlate GPS among men with favorable risk PCa. RETROSPECTIVE STUDY	Patients with NCCN very low and low risk PCa *n* = 296	GPS did not differ between quartile groups by any disease volume estimate at prostate biopsy or by PSAD.	In patients with NCCN very low and low risk PCA, GPS did not demonstrate meaningfully significant differences by disease volume at prostate biopsy. *SELECTION OF CANDIDATE FOR AS*
**Decipher** *(random biopsy)*	Kim et al. 2019 [83]	To assess a role for Decipher in predicting unfavourable disease. RETROSPECTIVE STUDY	Patients with NCCN very low/low risk or favorable-intermediate risk PCa and who received RP as first treatment. *n* = 266	In MVA when adjusting for CAPRA, Decipher was an independent predictor of AP.	Decipher can be applied to prostate biopsies from NCCN very-low/low and favorable-intermediate risk patients to predict AP found in prostatectomy pathology that would make a patient an inappropriate candidate for AS. *SELECTION OF CANDIDATE FOR AS*
**Decipher** *(random biopsy)*	Herlemann et al. 2020 [84]	To evaluate Decipher’s prognostic ability to predict unvafouvarble disease (defined as GG 3−5, pT3b or higher, or lymph node invasion (LNI)) at RP within the NCCN favorable intermediate risk group while accounting for clinical risk using the linear, extensively validated CAPRA score. RETROSPECTIVE STUDY	Patients with NCCN favorable-intermediate risk PCa who received RP as first treatment. *n* = 647	Decipher was an independent predictor of unfavourable disease and remained significant when adjusting by CAPRA. Notably, favorable-intermediate risk with Decipher low or intermediate score did not associate with significantly higher odds of AP compared to very low/low risk.	Decipher may be useful for safely expanding the use of AS in NCCN favorable-intermediate risk group. *SELECTION OF CANDIDATE FOR AS*

AS: active surveillance; CPCs: Circulating prostate cells; DRE: digital rectal examination; GPS: genomic prostate score; GS: gleason score; GR: grade reclassification (upgrading); mpMRI: multiparametric magnetic resonance imaging; NCCN: national comprehensive cancer network; PCa: prostate cancer; PI-RADS: Prostate Imaging-Reporting and Data System; PRIAS: Prostate Cancer Research International Active Surveillance; PSA: prostate serum antigen; PSAD: prostate serum antigen density; RP: Radical prostatectomy.

**Table 4:** Biomarkers’ performance.

Biomarker	FDA approved	AS Selection	AS Monitoring
Serum biomarker			
Pro PSA and PHI	X	Yes	Yes
4KScore	X	Yes	No
Iso PSA		-	-
Circulating prostate cells		Yes	-
microRNA		Yes	-
Caveolin 1		Yes	-
Testosterone	Currently available	Yes	-
Stockholm3 test		?	?
Urine biomarker			
PCA3	X	Yes	No
TMPRSS2-ERG fusion		?	?
DNA methylation and miRNA		Preliminary study	-
Tissue biomarker			
OncotypeDx GPS©	No but commercially available	?	No
Decipher©	No but commercially available	Yes	-
Prolaris©	No but commercially available	-	-
Promark score©	No but commercially available	-	-

4K: 4 kallicrein; AS: active surveillance; DNA: deoxyribonucleic acid; FDA: Food and Drug Administration; GPS: genomic prostate score; PHI: Prostate Health Index; PCA3: Prostate cancer antigen 3; RNA: ribonucleic acid.

## Data Availability

Not applicable.

## Supplementary Materials

The following are available online at https://www.mdpi.com/article/10.3390/cancers13174251/s1, Table S1: Quality/Risk of Bias of the retrospective Included Studies.

## Abbreviation

4K	4 kallicrein
AS	Active Surveillance
CTC	Circulating Tumoral Cells
Cav-1	Caveolin-1
DRE	Digital Rectal Examination
GS	Gleason Score
mpMRI	Multiparametric Magnetic Resonance Imaging
NCCN	national comprehensive cancer network
PCa	Prostate Cancer
PCA3	Prostate Cancer Antigen 3
PHI	Prostate Health Index
PSA	Prostate Specific Antigen
RP	Radical prostatectomy
