# Supplementary material for: Biomarker in Active Surveillance for Prostate Cancer: A Systematic Review
Source: Cancers (Basel). 2021 Aug 24;13(17):4251. doi: 10.3390/cancers13174251 (PMC8428218; doi:10.3390/cancers13174251)
Supplement: Supplementary file 1 [file cancers-13-04251-s001.zip › cancers-1288446-supplementary.pdf]

Supplementary Materials

Biomarker in Active Surveillance for Prostate Cancer: A Systematic Review

Cécile Manceau, Gaëlle Fromont, Jean-Baptiste Beauval, Eric Barret, Laurent Brureau, Gilles Créhange, Charles Dariane, Gaëlle Fiard, Mathieu Gauthé, Romain Mathieu, Raphaële Renard-Penna, Guilhem Roubaud, Alain Ruffion, Paul Sargos, Morgan Rouprêt, Guillaume Ploussard and on behalf of the CC-AFU, Cancerology Committee of the Association Française d'Urologie

Table S1. Quality / Risk of Bias of the retrospective Included Studies.

	Retrospectives series *n*=14<br>Quality apraisal tool for case studyusing a modified Delphi technique	*n* (%)
Study population	1. Hypothesis/aim/objective stated clearly	14 (100%)
	2. Characteristics of the study participants described	14 (100%)
	3. Multicentre study	7 (50%)
	4. Inclusion and exclusion criteria explicit and appropriate	14 (100%)
	5. Participants recruited consecutively	12 (86%)
	6. Participants entering at a similar disease point	11 (78%)
Interventions	7. Intervention clearly described	14 (100%)
	8. Cointerventions clearly reported	14 (100%)
Outcomes	9. Outcome measures clearly defined	14 (100%)
	10. Relevant outcomes appropriately measured	14 (100%)
	11. Outcomes measured before and after the intervention	14 (100%)
Stats	12. Statistical tests appropriate to assess outcomes	14 (100%)
Results/ conclusion	13. Length of follow-up reported	14 (100%)
	14. Loss to follow-up reported	5 (36%)
	15. Estimates of the random variability in data analysis	14 (100%)
	16. Adverse events reported?	14 (100%)
Conflicts of interest	17. Concluions of the study supported	14 (100%)
	18.Competing interests and sources of support reported	14 (100%)
